# Cause and Treatment of Cerebro-Spinal Meningitis

**Published:** 1873-02

**Authors:** W. O’Daniel

**Affiliations:** Treasurer Georgia Medical Association, Bullard’s Station, Twiggs County, Georgia


### CAUSE AND TREATMENT OF CEREBRO-SPINAL MENINGITIS.

BY W. O'DANIEL, A.M., M.D.,

*Treasurer Georgia Medical Association, Bullard's Station, Twiggs County, Georgia.*

In September, 1867, the first cases of this fearful disease came under my professional care upon the plantation of Mr. Thomas Glover, in Twiggs county. His plantation is situated in a malarious section of the county, between two creeks. About equidistant from them (they not being more than three-quarters of a mile apart) a new settlement was made in the spring of the year. Several families of negroes were tenanted on this plantation. They, anticipating removal in the fall, went to work to erect temporary huts, which they only expected to occupy a few months. These huts were made of green pine saplings, without even removing the bark from them. Many of them after completion were not sufficiently high to admit a man of full size to stand erect without touching the roof; the floor being also made of the same material, laid upon the ground, and the spaces between them filled with clay and ordinary top soil. In these *miserable leaky* huts, built by themselves and of their own choice (I suppose to save labor and expense), in a section where no one ever escapes fever of a malarial type, they commenced their year's operations.

The worms soon made their way into these timbers; decay commenced; no facilities for ventilation except one narrow door (the spaces between the logs being chinked with mud);. the result was that early in the summer, chills and fever commenced upon them; they having previously enjoyed good health, as they had before lived in a healthy section of the county. These fevers continued during their stay at the newly-settled place, but generally yielded to the usual remedies indicated in bilious, intermittent and remittent fevers. But with the usual hepatic and splenitic complications, the sequelæ of these fevers, together with anæmia, general debility, and a disorded nervous system (all of which I attribute to malarious influence), they were attacked about the middle of October with the justly-dreaded and terrible disease, cerebro-spinal meningitis, mostly confined to males, and they *under* twenty years of age—several being about fifteen years of age. The weather was at that time very unfavorable, and most of those attacked were suffering with colds. I supposed that the cause of their *repeated* attacks of intermittent, remittent and bilious fevers was also the cause of meningitis, and I am still of the same opinion—such as malaria, changes in the temperature, over-exertion, exposure to wet and cold, frequent bathing at unseasonable hours, etc., but malaria of course the principal cause. Whilst I have seen a few isolated cases of this disease in my practice for several years, it has not occurred as an epidemic since 1867, which was confined to three localities, or I may have said plantations, not more than two or three miles distant from each other.

As regards treatment, I think the first and most important step is to deplete thoroughly, provided the condition of the patient will bear it with any degree of safety. Taking into consideration the idiosyncracy, etc., of the patient, I abstract blood from the arm freely, and employ heroic doses of calomel (say thirty grains), sinapisms to extremities, warm pediluvium, and thorough counter-irritation of spine. I at one time, in an extreme case, applied a heated iron from the occiput to the coccyx, I thought, with great benefit to my patient,—this completely blistered the spine its entire length,—and last, but not least, frequent and large doses of quinine, with a sufficiency of some preparation of opium to promote rest. If it can not be given per orem, I advise the hypodermic use of it. This course of treatment must, of course, be modified according to the malignity of the attack.

I do not think that every case of meningitis causes complete abolition of the senses; that peculiar glaring look over the shoulders; rigidity of the muscular system, especially those of the neck; head drawn back upon the spine; convulsions, perfect opisthotonos, etc. I therefore am of the opinion that we can have it in a mild form as well as other diseases. I see no good reason why we should not. I know we are slow to diagnosticate a case cerebro-spinal meningitis without most of the above symptoms—in fact, we cannot with any degree of certainty. But when it occurs as an epidemic, I think we have premonitory symptoms sufficient to put us on our guard, so that we may employ remedies which may prevent the most incurable forms of the disease. About one-fourth of the cases of the *very worst*

forms of the disease died, and I am quite confident that I have prevented many cases from arriving at that fearful and incurable stage.

I have frequently employed bromide of potassum in connection with the above, perhaps with good results, but I have never been able to get my consent to rely upon it alone, and therefore cannot extol it as some of my professional brethren do in the treatment of meningitis.

After convalescence, I advise tonics, and a strict adherence to the laws of hygiene.

